# Immune Cell Landscaping Reveals Distinct Immune Signatures of Inflammatory Bowel Disease

**DOI:** 10.3389/fimmu.2022.861790

**Published:** 2022-03-16

**Authors:** Xiaowu Bai, Weixin Liu, Hongxia Chen, Tao Zuo, Xiaojian Wu

**Affiliations:** ^1^ Department of Colorectal Surgery, The Sixth Affiliated Hospital, Sun Yat-sen University, Guangzhou, China; ^2^ Guangdong Institute of Gastroenterology, Guangdong Provincial Key Laboratory of Colorectal and Pelvic Floor Diseases, Supported by National Key Clinical Discipline, The Sixth Affiliated Hospital, Sun Yat-sen University, Guangzhou, China; ^3^ Department of Medicine and Therapeutics, Li Ka Shing Institute of Health Sciences, Chinese University of Hong Kong, Hong Kong, Hong Kong SAR, China

**Keywords:** IBD, inflammatory bowel disease, immune cell, landscape, adaptive immunity, innate immunity

## Abstract

Determining how the profile of immune cells varies with their disease subtypes and across lesion locations is critical for understanding the pathogenesis in inflammatory bowel disease (IBD), including Crohn’s disease (CD) and ulcerative colitis (UC). To that end, we herein combined the IBD TaMMA framework and the CIBERSORT pipeline to deconvolute the large amount of RNA-seq data from patients with IBD (both CD and UC were included) and healthy human controls across 28 cohorts (a total of 3,852 samples) while accommodating data heterogeneity across cohorts, to define the immune cell landscape of IBD. Our study uncovered that both absolute quantities of innate and adaptive immune cell populations were elevated in most intestinal regions of IBD patients, yet disease-specific (CD versus UC) and intestinal location (ileum, colon, and rectum)-specific features. In the ileum, the increase in innate immune cells was more pronounced in CD than UC. In contrast, innate and adaptive immune cells were elevated more drastically in the UC than CD in the rectum. Such revelation of immune signatures across the highly variable IBD phenotypes (in both disease subtypes and intestinal regions) underpins differential immune-pathophysiological mechanisms in IBD pathogenesis and therefore serves as a resource for the development of future targeted studies.

## Introduction

The incidence of inflammatory bowel disease (IBD) is rising in the twenty-first century (1). The exact etiology of IBD remains unclear. It is primarily thought to arise from an aggravated immune response towards the gut microbiota in genetically susceptible individuals (2). Initial activation of innate immunity provokes non-specific responses, and then, the continued stimulation of inflammation will activate adaptive immunity, which could lead to sustained chronic inflammation. Available evidence suggests that both dysregulated innate and adaptive immune pathways contribute to the aberrant intestinal inflammatory response in patients with IBD. Subtypes of IBD include ulcerative colitis (UC), which contiguously affects the colon, and Crohn’s disease (CD), which can present anywhere in the gastrointestinal tract. Different macroscopic patterns of inflammation may result from different patterns of immune response between CD and UC (3).

In the past decades, a large number of omics studies were deployed to understand the pathogenesis of IBD, particularly *via* the RNA-sequencing (RNA-seq) technology to pinpoint genes and cell compartments contributing to the disease course and phenotypes. However, due to the individual nature and small/modest sample sizes in each RNA-seq study (some studies only centered on a specific cell fraction), it hinders the power to find population-robust genes and cell types that are generic culprit to the pathogenesis and progression of IBD. Therefore, integrating the current existing RNA-seq datasets from publicly available studies, while minimizing batch effect with proper data harmonization, would largely facilitate this goal. Recently, multiple frameworks and pipelines were developed to integrate IBD datasets and to aid in cross-dataset RNA-seq analysis, including the IBD Transcriptome and Metatranscriptome Meta-Analysis (TaMMA) framework (4). The IBD TaMMA framework comprehensively collated the publicly available IBD RNA-seq (both Transcriptome and Metatranscriptome) datasets from IBD-derived and control samples across different tissues (4). In addition, CIBERSORT (Cell-type Identification By Estimating Relative Subsets Of RNA Transcripts) was recently developed to characterize immune cell compositions (including B cells, T cells, and innate immune cell subsets) in complex tissues by computing gene expression profiles (GEPs), which has a strong agreement with flow cytometry assessment of immune subsets in bulk tissues (5, 6). CIBERSORT was widely utilized in different complex tissues to analyze immune signatures (7, 8).

Here, we combined the IBD TaMMA framework and CIBERSORT to deconvolute the large amount of RNA-seq data from patients with IBD (both CD and UC—two IBD subtypes—were included) and healthy human controls across 28 cohorts (a total of 3,852 samples), to define the immune cell landscape of IBD. We identified and enumerated the composition of 22 immune cell types, which could be statistically more robust than individual studies alone. Our study uncovers both disease-specific (CD versus UC) and lesion location-specific immune cell features in IBD. Such large-scale analysis of immune signatures of IBD provides a comprehensive understanding of differential immune responses in IBD, therefore guiding precision immunotherapies for IBD in the future.

## Methods

### RNA-Sequencing Data Acquisition

**Figure 1:**
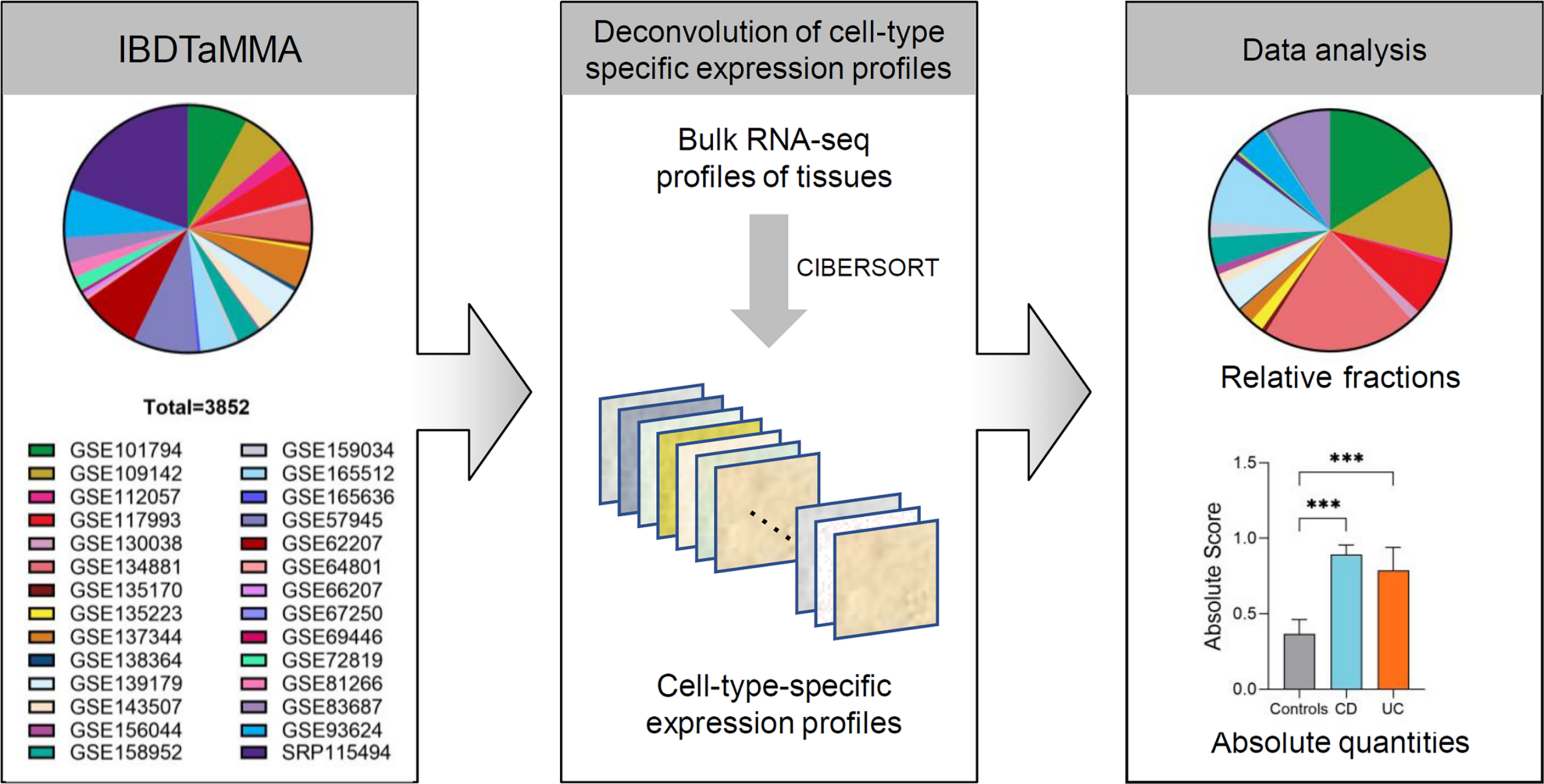
The analytic framework of immune cells profiling in patients with IBD and healthy individuals. Schematic depicting shows that CIBERSORT is applied to a multiple-cohort (n=28) gene expression profiles from bulk tissue transcriptomic data (a total of 3,852 subjects), comprising both patients with IBD and healthy individuals. Once immune cell fractions are determined, comparisons among different disease subtypes (CD and UC versus healthy individuals) across intestinal locations (ileum, colon, and rectum) are calculated with statistics. ***p < 0.001.

The Inflammatory Bowel Disease Transcriptome and Metatranscriptome Meta-Analysis (IBD TaMMA) framework contains publicly available gene expression files from 28 datasets of 26 independent studies before March 6, 2021 (4). All GEO/SRA numbers are given in **Figure 1**. GEPs of the 3,852 samples were batch corrected and merged to produce a mixture file by IBD TaMMA. We downloaded GEPs through OSFHOME (https://osf.io/yrxa7/).

### Identification of Immune Cells and Their Estimated Fractions

GEPs were prepared and formatted into one input file (also known as mixture file) according to the instructions in the manual on the website (http://cibersort.stanford.edu/tutorial.php). LM22 gene signature was downloaded through CIBERSORT website (https://cibersortx.stanford.edu/download.php). R version 4.1.1 was required to run CIBERSORT R script v1.04 (https://rdrr.io/github/zy26/SSMD/src/R/CIBERSORT_modified.R). The number of statistical permutations was set to 100 and quantile normalization was disabled. The absolute and relative modes of CIBERSORT were used to analyze data, respectively. The relative output was set so that the sum of all estimated immune-cell-type fractions for each sample equaled 1. Immune cell absolute estimates were calculated using their corresponding relative fractions. In CIBERSORT, the absolute mode converts estimated relative cellular fractions into a score that reflects the sample’s absolute proportion of each cell type. The median expression level of all genes in the signature matrix (LM22) divided by the median expression level of all genes in the sample yields the absolute immune score. Total innate immune cells score was calculated as a sum of NK cells (resting and activated), monocytes, macrophages (M0, M1, and M2), dendritic cells (resting and activated), mast cells (resting and activated), eosinophils, and neutrophils. Total B cells were calculated as a sum of naive B cells, memory B cells, and plasma cells. Total T cells were calculated as a sum of CD8+ T cells, naive CD4+ T cells, memory resting CD4+ T cells, memory-activated CD4+ T cells, follicular helper T cells, regulatory T (Treg) cells, and gamma delta (γδ) T cells.

### Statistical Analysis

Data are presented as mean ± standard error of the mean (SEM). Differences among the three groups were compared using Kruskal–Wallis non-parametric test, and multiple comparisons testing was performed by Dunn’s multiple comparisons test. All differences were considered statistically significant if *p* < 0.05. GraphPad Prism 8.0 and open-source R software (version 4.1.1) were used to perform statistical analysis.

### Data Availability

The immune profile datasets generated by CIBERSORT for each sample during the current study are available from the corresponding author on reasonable request.

## Results

### Overview of the Analytic Framework

A total of 28 source RNA-seq datasets from 26 independent studies were included from IBD TaMMA (consisting of 3,852 subjects, including 1,848 ileum, 448 colon, 480 rectum, and others). By CIBERSORT analysis, the relative fractions and absolute quantities of 22 immune cell types were calculated from all samples. We then characterized the immune cell landscape and signatures in patients with IBD (across disease subtypes and intestinal lesion locations) compared to that in healthy individuals (**Figure 1**).

### Landscape of Relative Fractions of Immune Cell Populations in IBD

**Figure 2:**
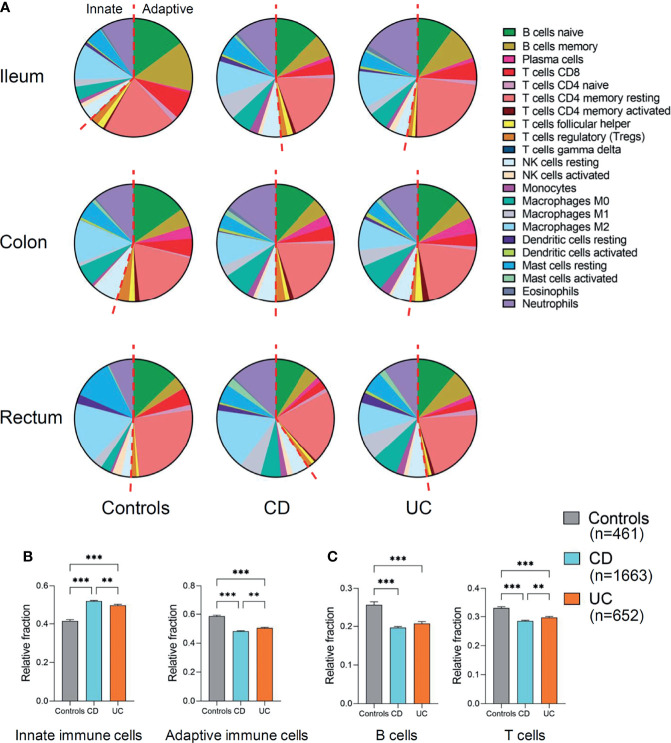
Relative fraction of immune cells profiling in patients with IBD and healthy individuals. **(A)** Relative fractions of immune cells profiling across three intestinal locations (ileum, colon, and rectum). The left of red dashed line represents innate immune cells and the right of the dashed line represents adaptive immune cells. **(B)** Comparisons of the total innate immune cells and adaptive immune cells (ileum, colon, and rectum combined) among three disease subtypes. **(C)** Comparisons of the total B and T cells (ileum, colon, and rectum combined) among three disease subtypes. Statistical significance was determined by Kruskal–Wallis non-parametric test and Dunn’s multiple comparisons test. ^**^ *p* < 0.01, ^***^ *p* < 0.001.

First, we compared the relative fractions of 22 immune cell types in different disease subtypes (CD and UC versus controls) and across different intestinal locations (ileum, colon, or rectum) (**Figure 2A**). Overall, the proportion of adaptive immune cells was decreased across the three intestinal locations (ileum, colon, and rectum) of CD and UC as compared to those of healthy controls, while the proportion of innate immune cells was correspondingly increased (**Figure 2A**). This alteration indicated the imbalance of immune system across different intestinal locations in CD and UC. The cumulative abundance (relative abundance) of innate immune cells (ileum, colon, and rectum combined) was significantly higher in both CD and UC, compared with that in healthy controls (both *p* < 0.001, **Figure 2B**). In contrast, the cumulative abundance (relative abundance) of adaptive immune cells was decreased in both CD and UC, compared with healthy controls (both *p* < 0.001, **Figure 2B**). Then, we particularly investigated the proportion of the adaptive immune cell subtypes, namely, B and T cells, in patients with IBD versus healthy controls. The relative fractions of the B- and T-cell populations were both decreased in CD and UC, compared with those in healthy controls (all *p* < 0.001, **Figure 2C**). These data together suggest a decrease in the relative fraction of adaptive immune cells (including B and T cells) concomitant with an increase in the relative fraction of innate immune cells in both CD and UC, compared to healthy individuals.

### Elevated Absolute Quantities of Innate and Adaptive Immune Cell Populations in IBD Patients

**Figure 3:**
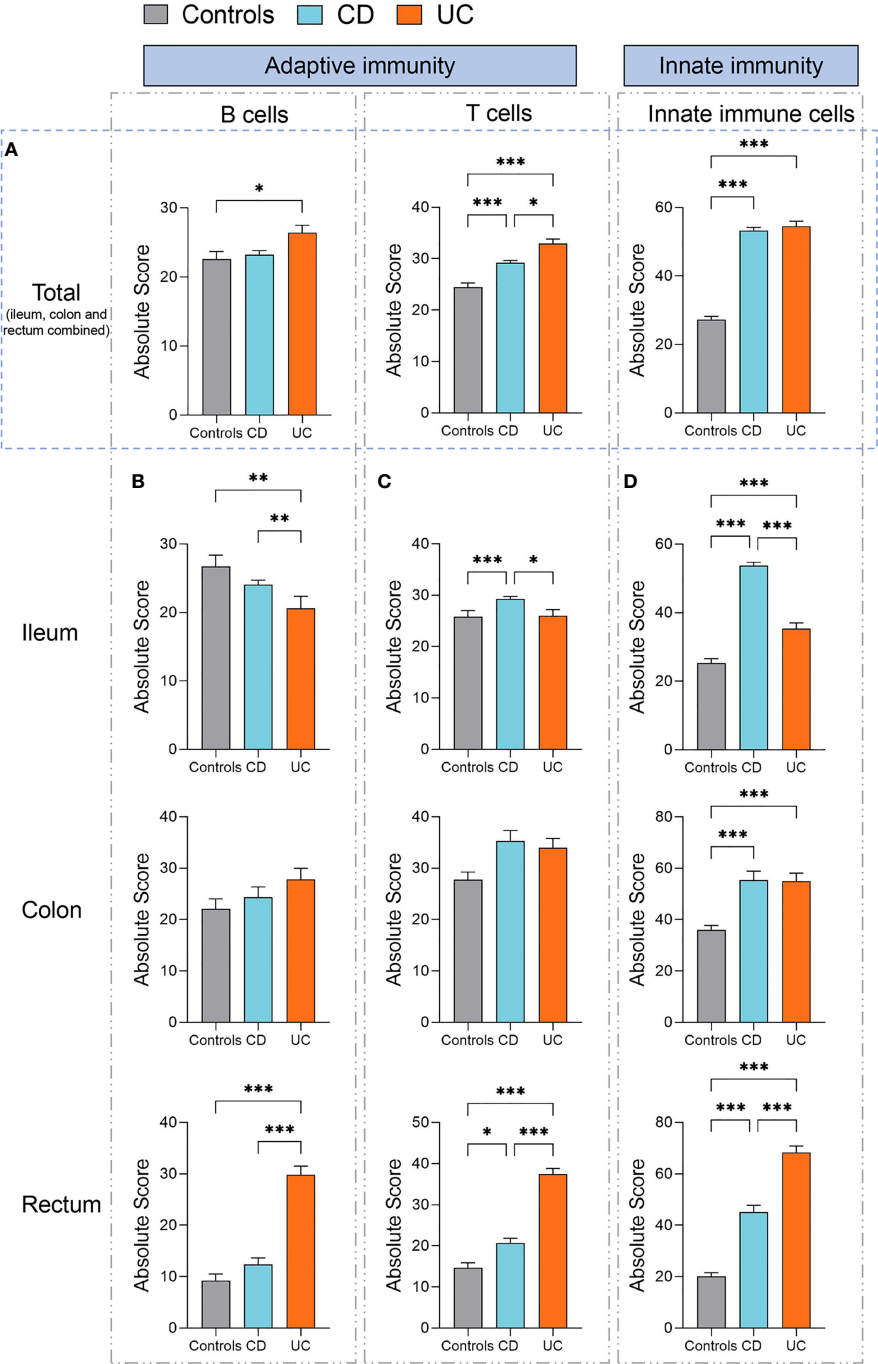
Absolute quantity of immune cells profiling in patients with IBD and healthy individuals. **(A)** Comparisons of the total B cells, T cells, and innate immune cells (ileum, colon, and rectum combined) among three disease subtypes (healthy controls, n=461; CD, n=1663; UC, n=652). **(B)** Comparisons of the total B cells among three disease subtypes in the ileum, colon, and rectum respectively. **(C)** Comparisons of the total T cells among three disease subtypes in the ileum, colon, and rectum respectively. **(D)** Comparisons of the total innate immune cells among three disease subtypes in the ileum, colon, and rectum, respectively. Statistical significance was determined by Kruskal–Wallis non-parametric test and Dunn’s multiple comparisons test. *p < 0.05, **p < 0.01, ***p < 0.001.

To gain a fine-scale insight into the alterations in the immune cell populations in IBD versus healthy controls, we additionally analyzed the absolute quantities of 22 immune cell types, as measured by CIBERSORT in absolute score for each immune cell population. We found that the absolute quantities of B cells, T cells, and innate immune cells were all significantly increased in UC (ileum, colon, and rectum combined) compared to healthy controls (*p* < 0.05, *p* < 0.001, and *p* < 0.001, respectively, **Figure 3A**), while only the absolute quantities of T cells and innate immune cells (ileum, colon, and rectum combined) were observed to be significantly increased in CD compared to healthy controls (both *p* < 0.001, **Figure 3A**). Moreover, the absolute quantity of T cells (ileum, colon, and rectum combined) was significantly higher in UC than CD (*p* < 0.05, **Figure 3A**). These data suggest that IBD has disease-specific immune features between CD and UC.

Next, we specifically assessed the absolute quantity variations in B cells, T cells, and innate immune cells across different intestinal lesion locations (ileum, colon, and rectum) in patients with CD or UC as compared to healthy controls.

The quantity of B cells was significantly increased in the rectum but decreased in the ileum of patients with UC, compared to healthy controls and patients with CD (all *p* < 0.01, **Figure 3B**). In comparison, the quantity of T cells was significantly increased in the ileum of CD than that of healthy controls and UC (*p* < 0.001 and 0.05, respectively, **Figure 3C**). Moreover, the quantity of T cells was significantly increased in the rectum of both CD and UC than that in healthy controls (*p* < 0.05 and 0.001, respectively, **Figure 3C**), while it was significantly higher in the UC rectum than that in the CD rectum (*p* < 0.001, **Figure 3C**).

We subsequently compared the quantity of innate immune cells across different intestinal lesion locations between CD, UC, and healthy controls. We found that the quantities of innate immune cells were all significantly increased in the ileum, colon, and rectum of both CD and UC patients, as compared to healthy controls (all *p* < 0.001, **Figure 3D**). The quantity of innate immune cells was significantly higher in the ileum of CD than that of UC (*p* < 0.001, **Figure 3D**), while this change was contrary to that observed in the rectum (*p* < 0.001, **Figure 3D**), hinting at intestinal location-specific alterations in the immune cell profile between CD and UC. Altogether, these data suggest that innate immune cells, B cells, and T cells are all elevated in most intestinal regions of CD and UC, yet they vary in a disease subtype and intestinal location-dependent manner.

### Immune Cell Profile Alterations in the Ileum of IBD Patients

**Figure 4:**
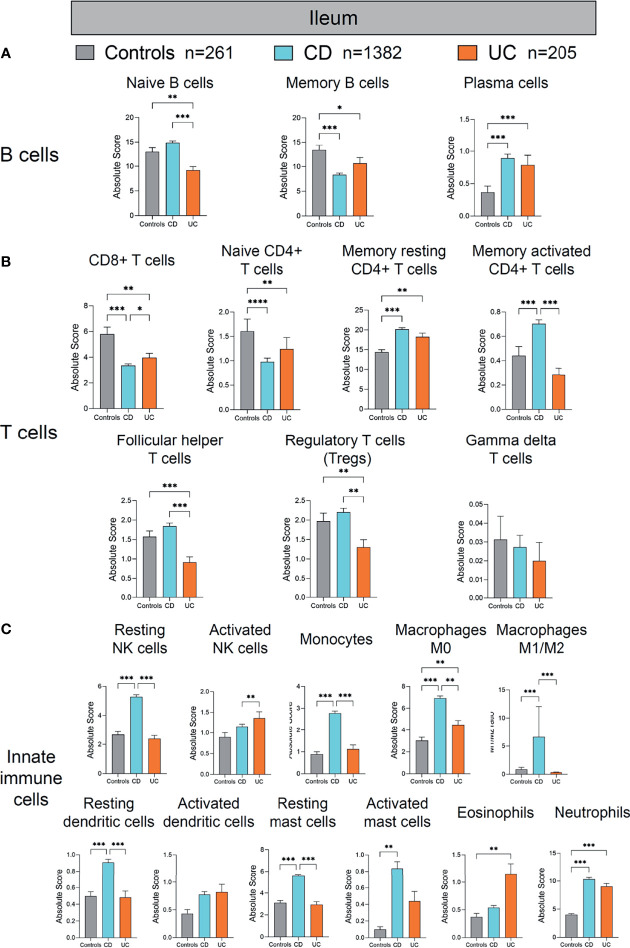
Absolute quantity of immune cells profiling in the ileum of patients with IBD and healthy individuals. **(A)** Comparisons of the B-cell subpopulations among three disease subtypes in the ileum. **(B)** Comparisons of the T-cell subpopulations among three disease subtypes in the ileum. **(C)** Comparisons of the innate immune cell subpopulations among three disease subtypes in the ileum. Statistical significance was determined by Kruskal–Wallis non-parametric test and Dunn’s multiple comparisons test. *p < 0.05; **p < 0.01, ***p < 0.001.

**Figure 5:**
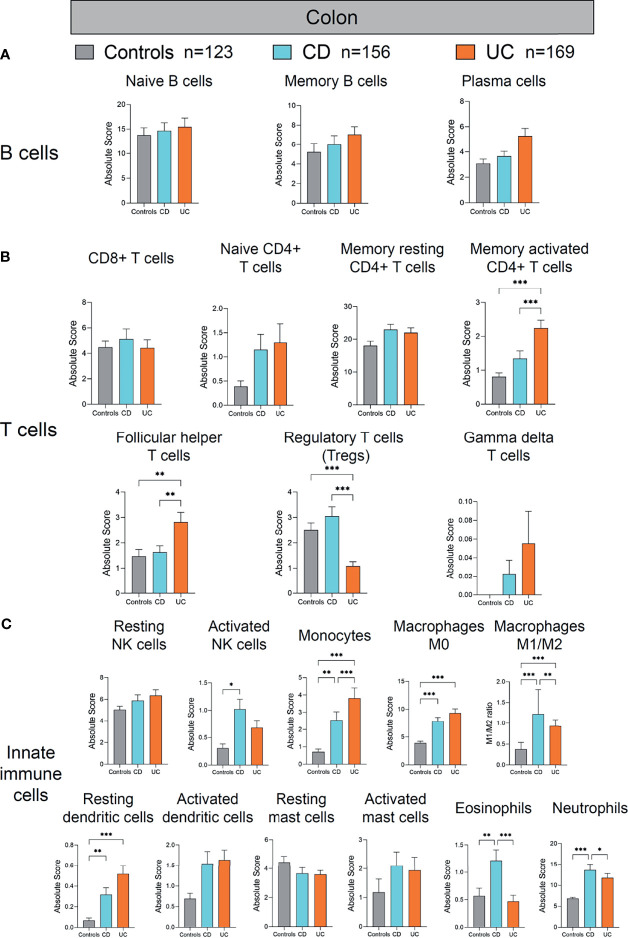
Absolute quantity of immune cells profiling in the colon of patients with IBD and healthy individuals. **(A)** Comparisons of the B-cell subpopulations among three disease subtypes in the colon. **(B)** Comparisons of the T-cell subpopulations among three disease subtypes in the colon. **(C)** Comparisons of the innate immune cell subpopulations among three disease subtypes in the colon. Statistical significance was determined by Kruskal–Wallis non-parametric test and Dunn’s multiple comparisons test. *p < 0.05; **p < 0.01, ***p < 0.001.

**Figure 6:**
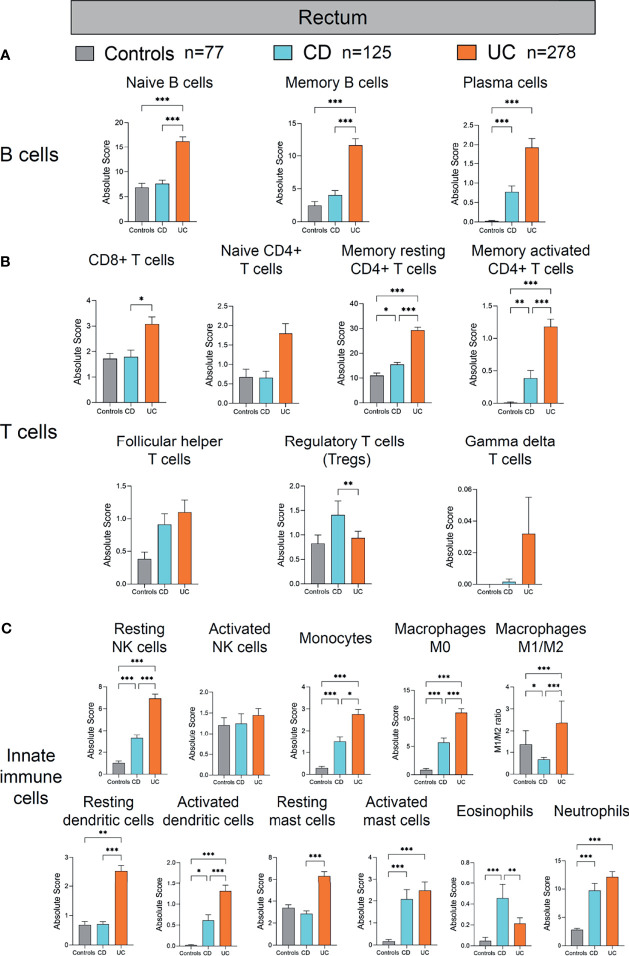
Absolute quantity of immune cells profiling in the rectum of patients with IBD and healthy individuals. **(A)** Comparisons of the B-cell subpopulations among three disease subtypes in the rectum. **(B)** Comparisons of the T-cell subpopulations among three disease subtypes in the rectum. **(C)** Comparisons of the innate immune cell subpopulations among three disease subtypes in the rectum. Statistical significance was determined by Kruskal–Wallis non-parametric test and Dunn’s multiple comparisons test. *p < 0.05, **p < 0.01, ***p < 0.001.

Given that the immune cell landscape exhibits location-specific features along the intestine axis (ileum, colon, and rectum), we then individually interrogated the alteration in each immune cell populations of the ileum (**Figure 4**), colon (**Figure 5**), and rectum (**Figure 6**), respectively, in IBD patients versus healthy controls.

First, we compared the immune cell profile of the ileum in CD, UC, and healthy controls (**Figure 4**). Although the absolute quantity of B cells in the ileum of UC was significantly decreased compared with healthy controls and CD (as shown in **Figure 3B**), different compartments of B cells (naive B cells, memory B cells, and plasma cells) showed discrepant alterations across the three groups (**Figure 4A** **)**. The quantity of naive B cells was significantly decreased in UC compared with healthy controls and CD (*p* < 0.01 and 0.001, respectively, **Figure 4A**). The quantity of memory B cells was significantly decreased in both CD and UC, compared with healthy controls (*p* < 0.001 and 0.05, respectively, **Figure 4A**). In contrast, the quantity of plasma cells, as main antibody-secreting B cells, was increased significantly in both CD and UC, compared to healthy controls (both *p* < 0.001, **Figure 4A**). The development trajectory of B cells originates from naive B cells, which later differentiate into plasma cells and memory B cells upon antigen recognition (9–12). These data indicate that more naive and memory B cells may transform to effector B cells in the ileum during IBD.

Next, we compared different T-cell populations [CD8+ T cells, naive CD4+ T cells, memory resting CD4+ T cells, memory activated CD4+ T cells, follicular helper T cells, Treg cells, gamma delta (γδ) T cells] in the ileum of CD and UC compared to healthy controls (**Figure 4B**). The quantity of CD8+ T cells was decreased significantly in CD and UC, compared with healthy controls (both *p* < 0.01, **Figure 4B**). The quantity of naive CD4+ T cells was significantly decreased, whereas that of memory resting CD4+ T cells was significantly increased in CD and UC, compared with healthy controls (all *p* < 0.01, **Figure 4B**). The quantity of memory-activated CD4+ T cells was increased significantly in CD, compared with healthy controls and UC (both *p* < 0.001, **Figure 4B**). The quantities of follicular helper T cells and Treg cells were both decreased significantly in UC, compared with healthy controls and CD (all *p* < 0.01, **Figure 4B**). Among these T-cell subpopulations, Treg cells are a specialized population acting to suppress immune response. The decrease in Tregs population in the ileum of UC patients versus healthy subjects indicates a lack of anti-inflammation mechanism in the ileum of UC, a disease mainly arising from colon and rectum inflammation (colon and rectum are the major inflicted intestinal region in UC). These data imply that the immune dysfunction may extend from distal intestine to proximal intestine. Overall, the largely heterogeneous alteration patterns in different ileal T-cell subpopulations between CD and UC compared to healthy controls are suggestive of a complicated, disease-specific T-cell dysfunction underlying disease pathogenesis, which warrants in-depth investigations.

We then investigated alterations in innate immune cells in the ileum of CD and UC patients versus healthy controls (**Figure 4C**). Among innate immune cells, resting NK cells, monocytes, macrophages M0, M1/M2 ratio, resting dendritic cells, and resting mast cells were all significantly increased in CD, compared with healthy controls and UC (all *p* < 0.01, **Figure 4C**). The quantity of activated mast cells was significantly increased in CD, compared with healthy controls (*p* < 0.01, **Figure 4C**). The quantity of neutrophils was increased significantly in CD and UC, compared with healthy controls (both *p* < 0.001, **Figure 4C**). The quantities of macrophages M0 and eosinophils were both increased significantly in UC, compared with healthy controls (both *p* < 0.01, **Figure 4C**). The quantity of activated NK cells was increased significantly in UC, compared with CD (*p* < 0.01, **Figure 4C**). These results together indicate that hyperactivation of ileal innate immune cells was more pronounced in CD than UC.

### Immune Cell Profile Alterations in the Colon of IBD Patients

Next, we analyzed the alterations of immune cells in the colon of patients with CD and UC compared to healthy controls (**Figure 5**). Among the studied subjects (CD, UC, and healthy controls), UC patients showed the most significant alterations in the colonic adaptive immune cell populations (including T- and B-cell subpopulations) (**Figures 5A, B**). Among the adaptive immune cells, colonic memory-activated CD4+ T cells and follicular helper T cells were significantly increased, whereas Treg cells were significantly decreased in UC, compared with healthy controls and CD (all *p* < 0.01, **Figure 5B**). Among the innate immune cells, activated NK cells were increased in the colon of CD, compared to healthy controls (*p* < 0.05, **Figure 5C**). Meanwhile, monocytes, macrophages M0, M1/M2 ratio, and resting dendritic cells were also increased significantly in the colon of CD and UC, compared to healthy controls (all *p* < 0.01, **Figure 5C**). Eosinophils and neutrophils were increased in the colon of CD, compared with healthy controls and UC (all *p* < 0.05, **Figure 5C**). Overall, compared to the immune cell profile alterations in the ileum in IBD versus healthy controls, the alterations in colonic immune cell profile were rather modest in IBD versus healthy controls.

### Immune Cell Profile Alterations in the Rectum of IBD Patients

Lastly, we analyzed the alterations of immune cells in the colon of patients with CD and UC compared to healthy controls (**Figure 6**). Among the B-cell subpopulations, the naive and memory B-cell populations were both increased significantly in the rectum of UC, compared with healthy controls and CD (all *p* < 0.001, **Figure 6A**). Incidentally, the plasma cell population was increased significantly in the rectum of both CD and UC, compared with healthy controls (both *p* < 0.001, **Figure 6A**). These data together suggest a concordant expansion of B-cell subpopulations in the rectum of IBD patients, different from the mere expansion of the plasma B-cell subpopulation in the ileum of IBD patients (**Figure 4A**). Among the T-cell subpopulations, the memory resting CD4+ T cell and memory-activated CD4+ T cell populations were both increased significantly in the rectum of CD and UC, compared with healthy controls (all *p* < 0.05, **Figure 6B**). Moreover, CD8+ T cells and memory resting CD4+ T cells and memory activated CD4+ T cells were all increased significantly in the rectum of UC, compared with CD (*p* < 0.05 and *p* < 0.001, *p* < 0.001, respectively, **Figure 6B**). In contrast, Tregs were decreased significantly in the UC, compared with CD (*p* < 0.01, **Figure 6B**). Taken together, these results indicate that adaptive immune cell populations in the rectum were elevated in both CD and UC, yet more drastically in UC than CD.

With regard to innate immune cells, resting NK cells, monocytes, macrophages M0, activated dendritic cells, activated mast cells, and neutrophils were all increased significantly in the rectum of CD and UC, compared with healthy controls (all *p* < 0.05, **Figure 6C**). Resting dendritic cells and macrophages M1/M2 ratio were both significantly increased in the rectum of UC, compared with healthy controls and CD (all *p* < 0.01, **Figure 6C**). In comparison, eosinophils were increased significantly in the rectum of CD, compared with healthy controls and UC (*p* < 0.001 and 0.01, respectively, **Figure 6C**). These data suggest that different innate immune cell populations were upregulated in CD and UC, potentially playing critical roles in the disease phenotype and disease course. In addition, resting NK cells, monocytes, activated dendritic cells, and resting mast cells were all increased in the rectum of UC, compared with CD (all *p* < 0.05, **Figure 6C**). However, macrophages M1/M2 ratio were decreased in the rectum of CD, compared with healthy controls (*p* < 0.05, **Figure 6C**). Overall, similar to the alteration pattern in adaptive immune cells in the rectum of IBD patients versus healthy controls, elevated innate immune cells in the rectum were observed in both CD and UC, which was more pronounced in UC than CD.

## Discussion

IBD is postulated to result from immune dysregulation to environmental and microbial triggers in genetically susceptible individuals. However, the exact alterations in the immune landscape across the intestinal axis (particularly different intestinal segments) have been unclear in IBD, neither were the differences between CD and UC. Improved understanding of immune cell landscapes in intestinal tissues may shed light on new therapeutic targets in IBD that can be tailored to disease type (CD versus UC), location (ileum, colon, or rectum lesions), and even individual patients.

Here, we conducted to date the largest sample sized, across-cohort study profiling the enteric immune cells composition (in both relative fraction and absolute quantity) of patients with IBD in comparison to healthy individuals. The adaptive immune responses have previously been believed to play a dominant role in the pathogenesis of IBD. However, due to recent advances in immunology and genetics, the innate immune responses are posited to be equally as important (if not more than that of adaptive immune responses) in inducing gut inflammation in IBD patients (13). Our findings confirmed this conception that both innate and adaptive immune cell populations were elevated in most intestinal regions of IBD patients. However, the elevation of innate immune cells seems higher than that of adaptive immune cells, resulting in that the relative fractions of adaptive immune cells (both B and T cells) decreased in the patients with IBD than healthy controls. Moreover, we identified both disease- and lesion location-specific immune signatures in IBD. For example, in the ileum, the increase in innate immune cells was more pronounced in CD than UC. In contrast, innate and adaptive immune cells were elevated more drastically in the UC than CD in the rectum. The rationale for our speculation is that it is somewhat related to illness characteristics and partially related to organ structure and function in various regions. For example, UC affects mainly the colon, but CD can affect any part of the GI tract, from the mouth to the anus, but is typically limited to the small intestine, particularly the terminal ileum. The main purpose of the small intestinal is to digest and absorb food, whereas the colon’s main purpose is to hold stool.

Moreover, previous studies reported that B cells were enriched in IBD (3, 14–16). Our discovery of IBD-associated B cells adds to and expands on this understanding. Plasma cells expanded both in the ileum and rectum of CD and UC, while expansion of memory B cells existed only in the rectum of UC and naive B cells increased in the rectum of UC and decreased in the ileum of UC. This suggests that, basically, B cells were hyperactivated in IBD, but we should distinguish phenotypes of their subsets carefully and explore its underlying mechanism in the future.

As regards to CD4+ T cells, resting memory CD4+ T cells increased both in the rectum and ileum of CD and UC, while activated memory CD4+ T cells increased in the rectum of both CD and UC, in the ileum of CD, and in the colon of UC, respectively, when compared to healthy controls. In addition, follicular helper T cells were increased in the ileum of CD and in the colon of UC conversely. These data suggest the disparate immune cell alterations between CD and UC across intestinal regions (17), which can be due to CD and UC both belonging to IBD but showing different behaviors. UC is limited to the colon. By contrast, CD can involve inflammation at any point of the GI tract from the mouth to the anus but is usually limited to the small intestinal, especially the terminal ileum.

The innate immune response represents our first line of defense against pathogens. It is non-specific and does not offer long-term immunity (memory), unlike the adaptive response (18–23). Innate immune cells, such as dendritic cells (DCs) and macrophages, can initiate rapid and effective inflammatory responses against microbial invasion. Our analysis suggest that many innate immune subsets were hyperactivated in tissues of CD and UC. For example, activated DCs were increased in the rectum of CD and UC, and resting DCs increased in the ileum of CD, in the rectum of UC, and in the colon of both CD and UC. Macrophage polarization occurs when macrophages respond to cues from their surroundings by adopting distinct functional programs. M1 and M2 are the two most prominent groups. Proinflammatory activity is performed by M1 macrophages. The M2, on the other hand, refers to macrophages that participate in constructive processes like wound healing and tissue repair, and those that produce anti-inflammatory cytokines like IL-10 to turn off damaging immune system activation. Our results demonstrated that the macrophage M1/M2 ratio was increased in the ileum of CD, in the rectum of UC, and in the colon of both CD and UC, which was consistent with previous studies (24, 25).

Understanding the distinct immune signatures across lesion locations in IBD is very helpful to design specific treatment strategies, especially when using biologics. For example, in ileal Crohn’s disease, the therapeutic benefits of cytokine blocking are confined to a minority of individuals. The reason for this is that a subset of patients had a distinct cellular module in inflamed tissues that included IgG plasma cells, inflammatory mononuclear phagocytes, activated T cells, and stromal cells, and its presence was linked to failure to achieve long-term corticosteroid-free remission after anti-TNF therapy (26). In the future, as a result of the rapid development and different specific functions of biologics, the majority of them should be used appropriately based on the immunological signatures of individual patients. Hence, our results provide the foundation of the development of precise biological therapies in IBD.

There are several limitations to our study, which necessitate a cautious interpretation of our findings. There is the issue of missing information regarding the severity of disease, which prevents us from distinguishing the characteristic of immune cells between inactive and active lesions. In addition, common treatments for IBD include biologics can affect the immune signatures of the intestinal in IBD. In addition, our model analyzed signatures of almost all subsets of immune cells but could not analyze specific subset deeply. Lastly, additional functional research is needed to determine whether our findings contribute to the disease development and severity.

In conclusion, we demonstrated that using CIBERSORT to deconvolve whole-tissue gene expression data yields refined information on the immune cell landscape of IBD. We showed that both innate and adaptive immunity hyperactivated in most intestinal regions of patients with IBD, yet disease-specific (CD versus UC) and intestinal location (ileum, colon, and rectum)-specific features. Such revelation of immune signatures across the highly variable IBD phenotypes (in both disease subtypes and intestinal regions) underpins differential immune-pathophysiological mechanisms in IBD pathogenesis and therefore serves as a resource for development of future targeted studies.

## Data Availability Statement

The datasets presented in this study can be found in online repositories. The names of the repository/repositories and accession number(s) can be found in the article/supplementary material.

## Author Contributions

XB was involved in study design, performed bioinformatics analysis, and drafted the manuscript. WL performed bioinformatics analysis. HC commented on the study. TZ and XW designed and supervised the study and revised the manuscript. All authors contributed to the article and approved the submitted version.

## Funding

This project was supported by research funds from National Natural Science Foundation of China (32100134 and 82172323).

## Conflict of Interest

The authors declare that the research was conducted in the absence of any commercial or financial relationships that could be construed as a potential conflict of interest.

## Publisher’s Note

All claims expressed in this article are solely those of the authors and do not necessarily represent those of their affiliated organizations, or those of the publisher, the editors and the reviewers. Any product that may be evaluated in this article, or claim that may be made by its manufacturer, is not guaranteed or endorsed by the publisher.

## Abbreviations

IBD, inflammatory bowel disease; CD, Crohn’s disease; UC, ulcerative colitis; GEPs, gene expression profiles; CIBERSORT, Cell-type Identification By Estimating Relative Subsets Of RNA Transcripts; TaMMA, transcriptome and metatranscriptome meta-analysis.
